# Long-term outcomes and quantitative radiologic analysis of extracranial–intracranial bypass for hemodynamically compromised chronic large artery occlusive disease

**DOI:** 10.1038/s41598-023-30874-8

**Published:** 2023-03-06

**Authors:** Hyunjun Jo, Si Un Lee, Han-Gil Jeong, Young-Deok Kim, Tackeun Kim, Leonard Sunwoo, Seung Pil Ban, Jae Seung Bang, Oki Kwon, Chang Wan Oh

**Affiliations:** 1 Department of Neurosurgery, Korea University Guro Hospital, Korea University College of Medicine, Seoul, Korea; 2 Department of Neurosurgery, Seoul National University Bundang Hospital, Seoul National University College of Medicine, 82 Gumi-ro 173 beon-gil, Bundang-gu, Seongnam-si, 13620 Gyeonggi-do Korea; 3 Department of Neurology, Seoul National University Bundang Hospital, Seoul National University College of Medicine, Seongnam-si, Korea; 4 Department of Radiology, Seoul National University Bundang Hospital, Seoul National University College of Medicine, Seongnam-si, Korea

**Keywords:** Medical research, Neurology

## Abstract

This study aimed to demonstrate the effectiveness of nonemergent extracranial-to-intracranial bypass (EIB) in symptomatic chronic large artery atherosclerotic stenosis or occlusive disease (LAA) through quantitative analysis of computed tomography perfusion (CTP) parameters using RAPID software. We retrospectively analyzed 86 patients who underwent nonemergent EIB due to symptomatic chronic LAA. CTP data obtained preoperatively, immediately postoperatively (PostOp0), and 6 months postoperatively (PostOp6M) after EIB were quantitatively analyzed through RAPID software, and their association with intraoperative bypass flow (BF) was assessed. The clinical outcomes, including neurologic state, incidence of recurrent infarction and complications, were also analyzed. The time-to-maximum (Tmax) > 8 s, > 6 s and > 4 s volumes decreased significantly at PostOp0 and up through PostOp6M (preoperative, 5, 51, and 223 ml (median), respectively; PostOp0, 0, 20.25, and 143 ml, respectively; PostOp6M, 0, 7.5, and 148.5 ml, respectively; *p* < 0.001, *p* < 0.001, and *p* < 0.001, respectively). The postoperative improvement in the Tmax > 6 s and > 4 s volumes was significantly correlated with the BF at PostOp0 and PostOp6M (PostOp0, r = 0.367 (*p* = 0.001) and r = 0.275 (*p* = 0.015), respectively; PostOp6M r = 0.511 (*p* < 0.001) and r = 0.391 (*p* = 0.001), respectively). The incidence of recurrent cerebral infarction was 4.7%, and there were no major complications that produced permanent neurological impairment. Nonemergent EIB under strict operation indications can be a feasible treatment for symptomatic, hemodynamically compromised LAA patients.

## Introduction

Although large artery atherosclerotic steno-occlusive disease (LAA) is a risk factor for primary or recurrent ischemic stroke, there is no clear treatment for prevention except medication^1,2^. Recently, endovascular treatment for LAA has been attempted via widening of the stenotic lesion, but intracranial arterial stenting does not fully overcome the periprocedural complications despite the development of various instruments, including improved catheters and stents^3–5^. On the other hand, although early randomized controlled trials (RCTs), such as the EC-IC Bypass Trial (EIBT) and the Carotid Occlusion Surgery Study (COSS), rejected extracranial-to-intracranial (EC-IC) bypass surgery^6–8^, its effectiveness has been re-evaluated, given recent indications of concerns with those studies^9–13^. In particular, many recent studies have shown that emergent EC-IC bypass (EIB) is effective in reducing stroke progression and recurrence in patients with acute ischemic stroke (AIS)^14–19^.

Furthermore, some studies have been conducted on nonemergent EIB surgery for preventing primary or recurrent stroke in hemodynamically compromised symptomatic LAA^20–22^. However, in most of these studies, either the number of subjects was small or quantitative analyses of perfusion changes, which could serve as objective evidence for the effectiveness of EIB, were not performed.

Therefore, we quantitatively analyzed pre- to postoperative changes in cerebral perfusion using computed tomography perfusion (CTP) with RAPID software and assessed their associations with clinical outcomes to determine the efficacy and safety of EIB for hemodynamically compromised symptomatic LAA.

## Materials and methods

### Patient enrollment

**Figure 1:**
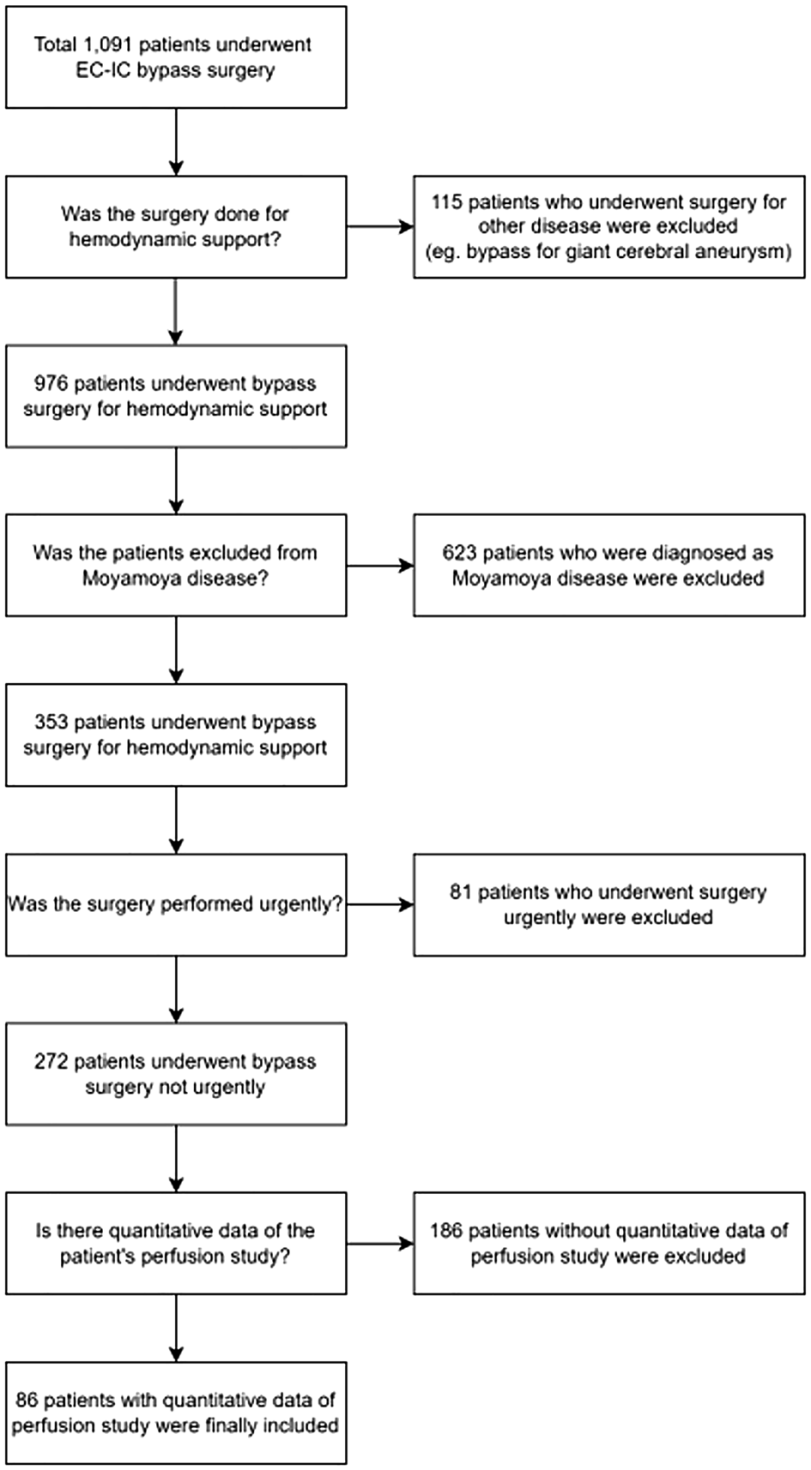
Flow diagram of the enrolled patients.

The medical data of patients who underwent nonemergent EIB for LAA between January 2006 and January 2020 were retrospectively reviewed under the approval of the Seoul National University Bundang Hospital institutional review board (IRB number: B-2103/673-103). The requirement to obtain informed consent from the patients has been waived by the Seoul National University Bundang Hospital institutional review board, and all methods were performed in accordance with the relevant guidelines and regulations. During the research period, we performed a total of 1,091 EIB procedures. A total of 738 patients who underwent EIB due to moyamoya disease or intracranial aneurysm or who underwent urgent or emergent bypass for AIS in the acute period or for acute ischemic symptoms such as transient ischemic attack (TIA) with an onset within 4 weeks were excluded. Among the remaining 272 patients, 186 whose perfusion data could not be reconstructed with RAPID software or who did not undergo pre- and immediate postoperative CTP scans were additionally excluded. Finally, 86 patients were analyzed in this study (Fig. 1).

### Operation indication

We performed nonemergent superficial temporal artery-to-middle cerebral artery (STA-MCA) bypass in patients with symptomatic hemodynamically compromised LAA who met all three of the following indications: 1) LAA at the intracranial artery or extracranial internal carotid artery (ICA), as diagnosed by computed tomography angiography (CTA), magnetic resonance imaging angiography (MRA) or transfemoral cerebral angiogram (TFCA); 2) moderate or severe perfusion delay, as confirmed by CTP; and 3) recurrent or aggravated TIA symptoms despite administration of the best medical treatment for at least 4 weeks. The definition of large artery included internal carotid artery, proximal middle cerebral artery (M1, M2), proximal anterior cerebral artery (A1, A2), vertebral artery, basilar artery, and proximal posterior cerebral artery (P1)^23^. Mild, moderate, and severe perfusion delay were defined as hypoperfusion with time-to-maximum (Tmax) > 4 s (s), Tmax > 6 s, and Tmax > 8 s, respectively^24^. For patients with AIS who had passed the acute phase without worsening of symptoms, EIB was performed if symptoms and perfusion do not improve or worsen despite medical treatment for at least 4 weeks to wait for collateral development^25–27^.

### Surgical procedure

The parietal or frontal branch of the patient's STA was dissected under general anesthesia. Craniotomy was performed around Chater's point where the angular, posterior temporal, or supramarginal arteries meet so that blood flow could be supplied to areas with severe perfusion delay while avoiding the eloquent area. The STA and distal MCA (M4) branches were anastomosed with 8–10 stiches with prolene 10–0 sutures. After anastomosis, the STA flow was measured using an ultrasonic flow meter. Because surgery was performed on patients whose symptoms persisted despite medical treatment, the operation was performed while maintaining aspirin monotherapy, which was restarted immediately after surgery. Intra-/post-operative blood pressure (BP) was maintained within 10% of the mean preoperative BP, and was generally controlled within the range of systolic BP 120–150 mmHg to prevent hypoperfusion or hyperperfusion.

### Radiologic analysis

All patients underwent CTP to evaluate the perfusion status within 1 week before surgery. To confirm short- and long-term improvement in perfusion, a follow-up perfusion study was performed within 48 h immediately after EIB and 6 months postoperatively, respectively. We quantitatively evaluated the CTP data of patients in two ways: one via comparison of preoperative and immediate postoperative perfusion data to ensure that the perfusion changes were due to the operation, and the other via the comparison of the data across the three time periods, i.e., preoperative, immediate postoperative, and 6 months postoperative, to confirm the long-term change in the perfusion status after bypass surgery. The CTP scan was taken using a 256 slice CT scanner (Brilliance, Philips Medical Systems, Best, The Netherlands) with a 40 mL bolus contrast injection and a 60- to 70 s CT scan of 8 cm of brain tissue. Image data is sent to RAPID software (version 5.0.4, iSchemaView, Menlo Park, California, USA) after capturing the entire passage of contrast through the brain with the scan cycles every 1–3 s. The Tmax > 10 s, Tmax > 8 s, Tmax > 6 s, and Tmax > 4 s of the time-to-peak (TTP) were analyzed using RAPID. Cerebral blood flow (CBF) < 30%, which is known to represent the ischemic core volume, and the difference between Tmax > 6 s and CBF < 30%, which is known to indicate salvageable tissue, that is, the mismatch volume or penumbra, were also compared.

All patients underwent postoperative diffusion MRI between 1 and 3 days after bypass, and additional MRI scan were performed when neurological symptoms occurred. One week after surgery, TFCA was performed to confirm whether the bypass pedicle was intact.

### Clinical outcomes

To evaluate the effectiveness of EIB, the occurrence of primary or recurrent infarction and TIA was checked. Infarction was defined as having neurologic symptoms and diffusion restriction at the site at which it was localized on diffusion MRI, and a scatter diffusion restriction point less than 3 mm on postoperative diffusion MRI without any neurological symptoms was defined as nonsignificant silent infarction, which was expected to be caused by transient hemodynamic instability or microembolism formed during anastomosis^28^. In addition to infarction, we also analyzed the changes in TIA symptoms after surgery. The above points were analyzed by dividing the patients into those who had a previous infarction on the lesion side before surgery and those without a previous infarction.

### Statistical analysis

Statistical analysis was performed using IBM SPSS statistics 25 (SPSS, Chicago, Illinois). Continuous variables are expressed as the median (interquartile range). The Wilcoxon matched-pairs signed-rank test was performed to compare perfusion data between two time points, and the Friedman test was performed to compare the data across three time points. Pearson correlation analysis was performed to evaluate whether the intraoperative post-anastomosis BF and the degree of improvement in perfusion before and after EIB was significant considering that Pearson's coefficient of correlation (r) 0.3–0.5 is a low positive correlation, 0.5–0.7 is a moderate positive correlation, and 0.7–0.9 is a high positive correlation^29^. Improvement in both pre- and immediate postoperative perfusion status and preoperative and postoperative 6 month perfusion status were evaluated. Generally, a *p* value < 0.05 was considered statistically significant.

## Results

### Baseline characteristics

A total of 86 patients who underwent nonemergent EIB for LAA were included in this study. The median age was 62 (51–68.3) years old, and 65 (75.6%) patients were male. Among the 86 patients, 61 (70.9%) patients had a history of previous AIS due to LAA, and 40 (46.5%) patients showed recurrent or aggravated TIA. A median of 3.0 (1.2–7.5) months transpired from the time of diagnosis of the first AIS or the onset of the first TIA symptoms to the time of surgery, and the follow-up period after surgery was 46.3 (33.5–67.4) months, i.e., patients with a follow-up period of at least approximately 3 years were analyzed. Fifty-four patients (62.8%) and 32 patients (37.2%) underwent EIB due to arterial occlusion and arterial stenosis, respectively. Additionally, the locations of the lesion and the preoperative modified Rankin Scale scores are shown in Table 1.

**Table 1:** Patient characteristics.

Variables	Total	Previous infarction	Without infarction	*p*-value
Total, number (%)	86 (100)	61 (70.9)	25 (29.1)	
Age*	62 (51–68.25)	62.5 (51–68)	59 (43–68)	0.958
Sex (male, number (%))	65 (75.6)	48 (78.7)	17 (68.0)	0.295
Medical history, number (%)				
Hypertension	61 (70.9)	46 (75.4)	15 (60.0)	0.153
Diabetes mellitus	26 (30.2)	20 (32.8)	6 (24.0)	0.420
Hyperlipidemia	27 (31.4)	22 (36.1)	5 (20.0)	0.145
TIA (number (%))	40 (46.5)	18 (29.5)	25 (100.0)	0.000
Symptom to operation, months*	2.97 (1.2–7.47)	2.87 (1.085–6.3525)	4.73 (1.63–12)	0.154
Follow-up period, months*	46.25 (33.47–67.37)	45.88 (33.6025–67.31)	46.27 (33.1–67.37)	0.902
Side (right, number (%))	38 (44.2)	30 (49.2)	8 (32.0)	0.145
Location of lesion, number (%)			0.490	
Proximal ICA	32 (37.2)	23 (37.7)	9 (36.0)	
Distal ICA	25 (29.1)	19 (31.1)	6 (24.0)	
M1	26 (30.2)	18 (29.5)	8 (32.0)	
M2	3 (3.5)	1 (1.6)	2 (8.0)	
Lesion severity, number (%)			0.096	
Occlusion	54 (62.8)	42 (68.9)	12 (48.0)	
Severe stenosis	32 (37.2)	19 (31.1)	13 (52.0)	
Pre-operative mRS, number (%)			0.001	
0	8 (9.3)	4	4	
1	39 (45.3)	20	19	
2	20 (23.3)	18	2	
3	11 (12.8)	11	0	
4	6 (7.0)	6	0	
5	2 (2.3)	2	0	
STA flow at operation field (ml/s)*	40 (27–58)	40.5 (24.5–69)	40 (27–56)	0.830
PreOp RAPID				
Tmax > 10 s	0 (0–5)	0 (0–5.75)	0 (0–5)	0.370
Tmax > 8 s	3.5 (0–41.25)	4 (0–47)	4 (0–15)	0.270
Tmax > 6 s	48 (5.75–139.75)	56.5 (11–151.25)	32 (5–131)	0.219
Tmax > 4 s	216 (116.5–315.25)	232 (125.25–317)	210(79–287)	0.260
Mismatch volume	48 (5–139.75)	56.5 (11–151.25)	32 (5–131)	0.233
PostOp 0 RAPID				
Tmax > 10 s	0 (0–3)	0 (0–3)	0 (0–0)	0.185
Tmax > 8 s	0 (0–9)	3 (0–16)	0 (0–0)	0.013
Tmax > 6 s	16 (0–70)	29.5 (4.25–84.75)	5 (0–22)	0.008
Tmax > 4 s	141.5 (83.75–266)	168 (96.25–278)	108 (51–180)	0.028
Mismatch volume	12.5 (0–66)	28.5 (0.5–78.25)	1 (0–21)	0.011

TIA, transient ischemic attack; ICA, internal carotid artery; mRS, modified Rankin Scale; STA, superficial temporal artery; PreOp, pre-operative; PostOp 0, immediate post-operative.

*Median (interquartile).

### Radiologic analysis

#### Quantitative assessment of immediate postoperative perfusion status

All 86 patients underwent TFCA within 1 week after EIB, and bypass occlusion was confirmed in 2 (2.3%) patients. We analyzed the CTP data reconstructed with RAPID software of all 86 patients. When comparing the group with previous infarction and the group without previous infarction, there was no significant difference in the preoperative Tmax > 10 s, Tmax > 8 s, Tmax > 6 s, Tmax > 4 s volumes and mismatch. However, the immediate postoperative Tmax > 8 s (*p* = 0.013), Tmax > 6 s (*p* = 0.008), Tmax > 4 s volumes (*p* = 0.028) and mismatch (*p* = 0.011) were significantly lower in the group without previous infarction (Table 1).

Before and immediately after surgery, Tmax > 10 s showed no significant difference (0 ml (0–5) vs. 0 ml (0–3), respectively, *p* = 0.510). Tmax > 8 s showed a significant difference (3.5 ml (0–41.25) vs. 0 ml (0–9)) before and after surgery, respectively (*p* = 0.019). Tmax > 6 s (48 ml (5.75–139.75) to 16 ml (0–70)) and Tmax > 4 s (216 ml (116.5–315.25) to 141.5 ml (83.75–266)) were also significantly different, with p values of 0.001 and < 0.001, respectively. CBF < 30% did not show a significant difference from 0 ml (0–0) before surgery to 0 ml (0–0) after surgery (*p* = 0.177). The mismatch volume, that is, the penumbra volume, calculated as the difference between Tmax > 6 s and CBF < 30%, showed a statistically significant improvement from 48 ml (5–139.75) before surgery to 12.5 ml (0–66) after surgery (*p* < 0.001) (Table 2).

**Table 2:** Short- and long-term comparison of CTP data (median (interquartile)).

Short term comparison of CTP quantitative data (n = 86)				
CTP parameter	PreOp	PostOp 0	p-value	
Tmax > 10 s (ml)	0 (0–5)	0 (0–3)	0.510	
Tmax > 8 s (ml)	3.5 (0–41.25)	0 (0–9)	0.019	
Tmax > 6 s (ml)	48 (5.75–139.75)	16 (0–70)	0.001	
Tmax > 4 s (ml)	216 (116.5–315.25)	141.5 (83.75–266)	< 0.001	
CBF < 30% (ml)	0 (0–0)	0 (0–0)	0.177	
Mismatch volume (ml)	48 (5–139.75)	12.5 (0–66)	< 0.001	

Long term comparison of CTP quantitative data (n = 74)				
CTP parameter	PreOp	PostOp 0	PostOp 6 M	*p*-value
Tmax > 10 s (ml)	0 (0–5.25)	0 (0–3)	0 (0–0)	0.002
Tmax > 8 s (ml)	5 (0–43)	0 (0–12.25)	0 (0–4.25)	< 0.001
Tmax > 6 s (ml)	51 (8.25–145.25)	20.25 (0–73.5)	7.5 (0–42.25)	< 0.001
Tmax > 4 s (ml)	223 (116.5–321.75)	143 (86.5–266)	148.5 (52.25–323)	< 0.001
CBF < 30% (ml)	0 (0–0)	0 (0–0)	0 (0–0)	0.250
Mismatch volume (ml)	51 (5.75–145.25)	19 (0–68.25)	7 (0–42.25)	< 0.001

CBF, cerebral blood flow; CTP, perfusion computed tomography; preOp, preoperative; postOp 0, immediate postoperative; postOp 6 M, postoperative 6 months; Tmax, time-to-maximum.

#### Quantitative assessment of long-term postoperative perfusion status

**Figure 2:**
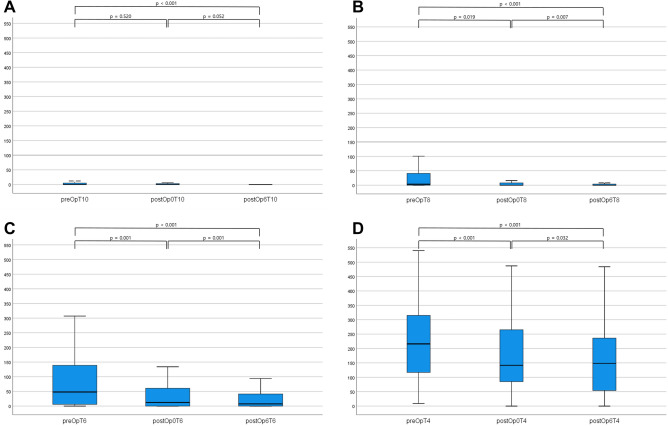
(**A**) The value of Tmax > 10 s continuously decreased as follows: preoperatively, 0 ml (0–5.25); immediately postoperatively, 0 ml (0–3); and 6 months postoperatively, 0 ml (0–0) (*p* = 0.002). (**B**) The value of Tmax > 8 s continuously decreased as follows: preoperatively, 5 ml (0–43); immediately postoperatively, 0 ml (0–12.24); and 6 months postoperatively, 0 ml (0–4.25) (*p* < 0.001). (**C**) The value of Tmax > 6 s continuously decreased as follows: preoperatively, 51 ml (8.25–145.25); immediately postoperatively, 20.25 ml (0–73.5); and 6 months postoperatively, 7.5 ml (0–42.25) (*p* < 0.001). (**D**) The value of Tmax > 4 s continuously decreased as follows: preoperatively, 223 ml (116.5–321.75); immediately postoperatively, 143 ml (86.5–266); and 6 months postoperatively, 148.5 ml (52.25–323) (*p* < 0.001).

Of the patients analyzed above, the 6-month postoperative perfusion data of 74 patients could also be analyzed. Tmax > 10 s before surgery, immediately after surgery, and 6 months after surgery showed a significant decreasing trend, from 0 ml (0–5.25) to 0 ml (0–3), and 0 ml (0–0), respectively (*p* = 0.002). Tmax > 8 s continued to show a significant decrease from 5 ml (0–43) to 0 ml (0–12.25) and 0 ml (0–4.25) (*p* < 0.001), and Tmax > 6 s also showed a significant decreasing trend from 51 ml (8.25–145.25) to 20.25 ml (0–73.5) and 7.5 ml (0–42.25) (*p* < 0.001). Tmax > 4 s also showed significant changes, from 223 ml (116.5–321.75) to 143 ml (86.5–266) and 148.5 ml (52.25–323) (*p* < 0.001). However, CBF < 30% did not show a significant change, from 0 ml (0–0) to 0 ml (0–0) and 0 ml (0–0) (*p* = 0.250). Finally, the penumbra volume calculated by the abovementioned method showed statistically significant changes, from 51 ml (5.75–145.25) to 19 ml (0–68.25) and 7 ml (0–42.25) (*p* < 0.001). The Wilcoxon matched-pairs signed-rank test was performed to determine whether there was a significant difference in perfusion status before and immediately after surgery, immediately after surgery and 6 months after surgery, and before and 6 months after surgery; the corresponding p values are shown in Fig. 2 and Table 2.

### Clinical outcomes

During the 3-year mean observational period for the 86 patients, only 4 (4.7%) patients with recurrent cerebral infarction were identified, and there was no primary infarction. In 2 (2.3%) of 4 patients with recurrent infarction, infarction occurred on the 5th and 7th days after bypass, and 1 of those 2 patients showed bypass occlusion. The other 2 (2.3%) of 4 patients with recurrent infarction presented with infarction on the 63rd and 352th days after bypass, respectively (Table 3). Insignificant silent infarction occurred in 5 patients (5.8%), 2 of whom had previous infarction and 3 of whom did not. Seven patients (8.1%) showed TIA symptoms without infarction, but all TIA symptoms disappeared within 1 month after EIB. It is noteworthy that 2 out of 4 patients who developed cerebral infarction were patients with bypass failure. That is, among 84 patients with an intact bypass pedicle, the occurrence of cerebral infarction was 2.4% (2/84).

**Table 3:** Details of the 4 patients with postoperative recurrent cerebral infarction.

Age	Sex	Initial presentation	Location	Severity	Onset to operation (months)	Operation to infarction (days)	Intraoperative bypass flow (ml/min)	Bypass flow on angiogram	Pre-operative mRS	Post-operative mRS	Pre-operative Tmax > 6 s (ml)	post-operative 0 Tmax > 6 s (ml)
M	47	Infarction	Rt. pICA	Occlusion	3.7	5	30	Occlusion	3	2	31	94
M	41	Infarction	Lt. M1	Stenosis	6.4	352	28	Patent	1	0	12	0
M	62	Infarction + TIA	Rt. dICA	Occlusion	6.2	63	28	Occlusion	1	0	13	24
M	69	Infarction	Lt. pICA	Occlusion	0.9	7	63	Patent	3	1	54	8

Operation site infections occurred in 4 (4.7%) patients. After antibiotic treatment, 3 patients improved, and 1 patient underwent empyema removal. Three (3.5%) patients had a seizure event within 3 days after surgery, but the condition did not persist thereafter. Four (4.7%) patients had chronic subdural hematoma at the operation site; 3 patients improved after burr-hole drainage, and 1 patient improved after conservative treatment. Overall, there were no major complications that left permanent neurological impairment (Table 4).

**Table 4:** Post-operative non-neurologic complications after EC-IC bypass surgery (number (%)).

Operation site infection	4 (4.7)
Seizure	3 (3.5)
Operation site chronic subdural hematoma	4 (4.7)

EC-IC, extracranial-to-intracranial; TIA, transient ischemic attack.

### Correlation between BF and perfusion parameter

**Figure 3:**
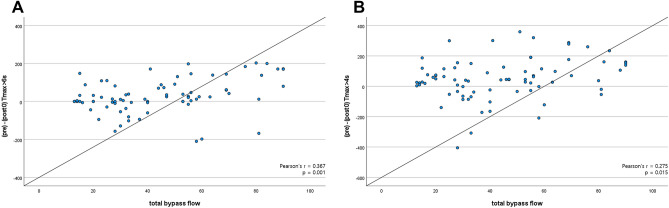
(**A**: The amount of change in Tmax > 6 s pre- and immediately postoperatively was correlated with the amount of STA flow measured during surgery (Pearson's r = 0.367, *p* = 0.001). (**B**) The amount of change in Tmax > 4 s pre- and immediately postoperatively was correlated with the amount of STA flow measured during surgery (Pearson's r = 0.275, *p* = 0.015).

**Figure 4:**
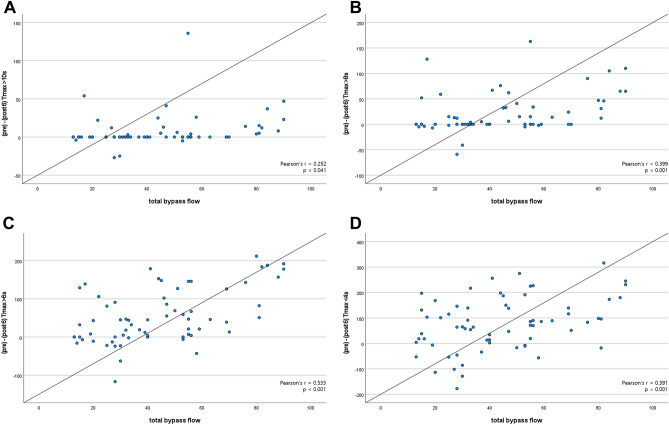
(**A**) The amount of change in Tmax > 10 s pre- and 6 months postoperatively was correlated with the amount of STA flow measured during surgery (Pearson's r = 0.252, *p* = 0.041). (**B**) The amount of change in Tmax > 8 s pre- and 6 months postoperatively was correlated with the amount of STA flow measured during surgery (Pearson's r = 0.399, *p* < 0.001). (**C**) The amount of change in Tmax > 6 s pre- and 6 months postoperatively was correlated with the amount of STA flow measured during surgery (Pearson's r = 0.533, *p* < 0.001). (**D**) The amount of change in Tmax > 4 s pre- and 6 months postoperatively was correlated with the amount of STA flow measured during surgery (Pearson's r = 0.391, *p* = 0.001).

Additional statistical analysis was performed to check whether there was a significant correlation between the BF as measured on STA after anastomosis and the degree of improvement in perfusion after surgery. First, Pearson correlation analysis was performed to determine whether the amount of change in the perfusion parameters before and immediately after surgery was related to BF. The amount of change in Tmax > 10 s, Tmax > 8 s, and CBF < 30% were not statistically significant (*p* = 0.242, 0.067, and 0.892, respectively) with a Pearson's r value of 0.135, 0.210, and 0.016, respectively. The changes in Tmax > 4 s were identified as having a p-value of 0.015, but Pearson's r value was 0.275, showing no positive correlation. However, the changes in Tmax > 6 s and penumbra volume showed a low positive correlation with Pearson's r values of 0.367 and 0.376, respectively (*p* = 0.001 and < 0.001, respectively) (Fig. 3). We then performed Pearson correlation analysis to evaluate the correlation between the BF and long-term change of perfusion parameters measured in the 6th month postoperatively. CBF < 30% still did not show a significant correlation, with Pearson's r at 0.006 (*p* = 0.959). Although the changes in Tmax > 10 s showed significant value (*p* = 0.041), Pearson's r showed no positive correlation (r = 0.252). However, the changes in Tmax > 8 s and Tmax > 4 s showed significant low positive correlations with Pearson's r values of 0.399 and 0.391, respectively (*p* < 0.001 and 0.001, respectively). The changes in Tmax > 6 s (r = 0.511; *p* < 0.001) and the penumbra volume (r = 0.537; *p* < 0.001) showed a significant moderate positive correlation, respectively (Fig. 4).

## Discussion

Ischemic stroke is one of the leading causes of death worldwide, and stroke recurrence worsens the prognosis of patients and increases both the patient's and society’s costs. Numerous studies have been conducted on the recurrence rate of ischemic stroke and treatment for the prevention of recurrence^2–33^. In particular, the incidence of recurrent ischemic stroke at 5 years in patients classified as LAA based on the Trial of Org 10172 in Acute Stroke Treatment (TOAST) classification was 8.2–16.8%^30^. However, a clear treatment for preventing recurrence of ischemic stroke due to LAA has yet to be established. As previously mentioned, several attempts to improve the disease through intracranial stent insertion in patients with intracranial stenosis have not been as successful as expected^3–5^. Conversely, there are some reports that nonemergent EIB, whose effectiveness had previously been questioned, is helpful in limited indications^20–22,34^.

### Nonemergent EIB for patients with symptomatic and hemodynamic compromised LAA

Two representative RCTs studied the effectiveness of nonemergent bypass for hemodynamically compromised LAA^35,36^. The EIBT analyzed the efficacy and safety of EIB in patients with TIA or stroke that had occurred within 3 months before entry into the study^35^. Perioperative ischemic events occurred in 12.2%, and major infarction occurred in 4.5%^35^. Furthermore, 18% and 20% of single strokes occurred in the medical treatment group and the EIB group, respectively, and two or more strokes occurred in 10% and 11% of each group over an average of 55.8 months, which demonstrated that EIB had no benefit in preventing stroke^35^. Another study was the COSS, which also compared a medical treatment group and EIB group, which consisted of ICA-occluded patients with TIA or ischemic stroke that had occurred within 120 days^36^. In that study, the 30-day rates for ipsilateral ischemic stroke were 14.3% in the EIB group and 2.0% in the medical treatment group, and the 2-year rates for the primary endpoint were 21.0% and 22.7%, respectively, indicating that EIB had no preventive effectiveness^36^. However, the problem with these two studies was that the perioperative morbidity was high, 12.2% and 14.3%, respectively^36^. Recently, as bypass technology has been developed and become widespread, several studies on EIB have reported good results with low perioperative morbidity^15–22,34,39–44^. Nevertheless, since most studies are on emergent EIB in AIS, they are of limited use in demonstrating the effectiveness and safety of nonemergent bypass for symptomatic and hemodynamically compromised LAA. We performed elective EIB for highly selected patients with symptomatic and hemodynamically compromised LAA, and cerebral infarction occurred in only 4.7% of patients over a mean period of 3 years without major complications associated with surgery, which is remarkably lower than the natural course of hemodynamically compromised LAA reported previously^2–33^.

Following quantitative analysis with RAPID software, we showed that cerebral perfusion could be improved and hemodynamic instability could be alleviated through EIB. Perfusion parameters before, immediately after surgery, and long-term indexes up to 6 months after surgery were compared sequentially, and the results demonstrated that most of the parameters improved after EIB. In particular, using imaging evidence, we established that EIB can prevent cerebral infarction, confirming that the volume of Tmax > 6 s, an index of penumbra^45^, is significantly improved. The recurrent TIA symptoms in all patients resolved within 1 month, and in patients with previous infarction, based only on if the operation was successful, symptoms disappeared in 97.6%; the other 2 of the 84 (2.4%) patients developed recurrent infarction. When nonemergent EC-IC bypass was performed on patients with symptomatic hemodynamically compromised chronic LAA under our indications, the surgical success rate was 97.7% (84/86), and the probability of symptom improvement was 97.6% (82/84).

### Comparison of perfusion parameters with or without previous infarction

When we compared patients with and without previous infarction, there were no significant differences in other characteristics, including preoperative perfusion parameters and intraoperative STA flow, with the exception of the preoperative mRS score. However, postoperative perfusion parameters were significantly better in the non-infarction group than in the previous infarction group. Although there is no known evidence for this, in the previous infarction group, it was assumed that the area where the infarction had already occurred would not have effectively increased perfusion. Additionally, since the previous-infarction group was more likely to have more severe arterial atherosclerosis, we also hypothesized that the intracranial arterial network, such as the leptomeningeal collateral flow, would be relatively poor, so less improvement in perfusion status may occur. Based on this, it can be hypothesized that in patients with symptomatic LAA, EIB before infarction may be more helpful in improving perfusion parameters.

### Relationship between BF and perfusion change

According to the results of this study, STA BF as measured with a flowmeter was positively correlated with the degree of perfusion improvement. Amin-Hanjani et al. demonstrated that the lower the cerebrovascular reserve capacity (CVRC), the higher BF and cut flow index (CFI; post-anastomosis BF (ml/min)/preoperative cut off flow (ml/min))^46^. Similarly, Inoue et al. demonstrated that the STA mean flow was significantly associated with regional cerebral blood flow when cerebral hemodynamics were evaluated by STA duplex ultrasonography^47^. Since CVRC is one of the factors that can affect BF, the lower the CVRC, the higher the BF, which can lead to more improved perfusion, that was assumed to be reflected in the results of this study. As a factor that can affect BF other than CVRC, it is advantageous to use a method that increases BF, and it may be helpful to select and design the STA and anastomosis site based on Poiseulle's law, which states that flow is proportional to the fourth power of diameter and inversely proportional to length. Accordingly, it is advantageous to select an STA or recipient artery with a larger diameter, although caution is required because there is a risk of hemorrhagic cerebral hyperperfusion syndrome if the STA is significantly thicker than the recipient artery^44–47^. Nakamizo et al. reported that the STA diameter and mean STA flow were significantly associated with CVRC, with an STA diameter cutoff value of 1.8 mm^48^. In addition, long-slit arteriotomy and fish-mouth cutting of the donor artery to enlarge the anastomosis site may be helpful^49,50^. Furthermore, according to Poiseulle's law, to reduce resistance, the length of the STA should be minimized to within the distance from the recipient artery as much as possible, which will contribute to increasing BF.

### Limitations

There are several limitations in this study. First, this study was conducted retrospectively. Additionally, it is a single-arm study without a control group. Therefore, in particular, there may be controversy in the indication of EIB for a small number of preoperative poor conditioned patients. To overcome these limitations, the effectiveness of EIB was evaluated quantitatively using CTP, and we compared the results indirectly with the results of other studies that demonstrated the natural course of symptomatic LAA. Second, we did not measure CVRC, one of the previously known indications of EIB. However, this study only included patients with symptomatic moderate to severe perfusion delay, and it is indirectly possible to predict a decrease in CVRC because they are all worse than stage I hemodynamic failure^51^. In addition, the area of Tmax > 6 s RAPID CTP is defined as penumbra in DIFUSE3, DAWN, and SWIFT PRIME trial, so it is considered reasonable enough as an indication of bypass^52–54^. Additionally, since this study was conducted in a single tertiary general hospital that has much experiences with bypass surgeries and postoperative care, there may be limitations in applying this result to all hospitals. However, as the bypass technique has recently improved and become more common, this treatment can be considered sufficient for other institutions.

## Conclusion

EIB in patients with symptomatic hemodynamically compromised LAA can contribute to lowering the incidence of recurrent infarction and improving perfusion parameters, which are positively related to intraoperative BF (Supplementary File 1).

## Supplementary Information

Supplementary Information.

## Data Availability

We provided all raw data used in this study as supplementary file.

## Abbreviations

LAA: Large artery atherosclerotic steno-occlusive disease
EC-IC: Extracranial-to-intracranial
EIB: EC-IC bypass
CTP: Computed tomography perfusion
Tmax: Time-to-maximum
STA-MCA: Superficial temporal artery-to-middle cerebral artery
TTP: Time-to-peak
CBF: Cerebral blood flow
mRS: Modified Rankin Scale
BF: Bypass flow
